# Flood-Ring Formation and Root Development in Response to Experimental Flooding of Young *Quercus robur* Trees

**DOI:** 10.3389/fpls.2016.00775

**Published:** 2016-06-14

**Authors:** Paul Copini, Jan den Ouden, Elisabeth M. R. Robert, Jacques C. Tardif, Walter A. Loesberg, Leo Goudzwaard, Ute Sass-Klaassen

**Affiliations:** ^1^Forest Ecology and Forest Management Group, Wageningen University and Research CentreWageningen, Netherlands; ^2^Alterra, Wageningen University and Research CentreWageningen, Netherlands; ^3^Laboratory of Wood Biology and Xylarium, Royal Museum for Central AfricaTervuren, Belgium; ^4^Laboratory of Plant Biology and Nature Management, Vrije Universiteit BrusselBrussels, Belgium; ^5^Centre for Forest Interdisciplinary Research, Department of Biology, The University of WinnipegWinnipeg, Canada

**Keywords:** flooding, hypoxia, leaf phenology, pedunculate oak, *Quercus robur*, vessel development, root development

## Abstract

Spring flooding in riparian forests can cause significant reductions in earlywood-vessel size in submerged stem parts of ring-porous tree species, leading to the presence of ‘flood rings’ that can be used as a proxy to reconstruct past flooding events, potentially over millennia. The mechanism of flood-ring formation and the relation with timing and duration of flooding are still to be elucidated. In this study, we experimentally flooded 4-year-old *Quercus robur* trees at three spring phenophases (late bud dormancy, budswell, and internode expansion) and over different flooding durations (2, 4, and 6 weeks) to a stem height of 50 cm. The effect of flooding on root and vessel development was assessed immediately after the flooding treatment and at the end of the growing season. Ring width and earlywood-vessel size and density were measured at 25- and 75-cm stem height and collapsed vessels were recorded. Stem flooding inhibited earlywood-vessel development in flooded stem parts. In addition, flooding upon budswell and internode expansion led to collapsed earlywood vessels below the water level. At the end of the growing season, mean earlywood-vessel size in the flooded stem parts (upon budswell and internode expansion) was always reduced by approximately 50% compared to non-flooded stem parts and 55% compared to control trees. This reduction was already present 2 weeks after flooding and occurred independent of flooding duration. Stem and root flooding were associated with significant root dieback after 4 and 6 weeks and mean radial growth was always reduced with increasing flooding duration. By comparing stem and root flooding, we conclude that flood rings only occur after stem flooding. As earlywood-vessel development was hampered during flooding, a considerable number of narrow earlywood vessels present later in the season, must have been formed after the actual flooding events. Our study indicates that root dieback, together with strongly reduced hydraulic conductivity due to anomalously narrow earlywood vessels in flooded stem parts, contribute to reduced radial growth after flooding events. Our findings support the value of flood rings to reconstruct spring flooding events that occurred prior to instrumental flood records.

## Introduction

Trees growing in riparian forests must cope with regular flooding events and may survive the anoxic conditions associated with flooding (Kozlowski, 1984; Siebel et al., 1998; Glenz et al., 2006). While flooding during dormancy may not affect trees, flooding during the growing season can severely affect development and growth (Gill, 1970; Kozlowski, 1984; Glenz et al., 2006). Species of oak (*Quercus*) and ash (*Fraxinus*) trees frequently occur along river systems in Europe (*Q. robur* L., *F. excelsior* L.), the United States of America, and Canada (e.g., *Q. macrocarpa* Michx., *Q. lyrata* Walter., *F. nigra* March., *F. pennsylvanica* March.). These species are ring porous and form large earlywood vessels in spring, followed by small latewood vessels later on in the growing season and have shown to be able to cope with 50 days of flooding as juveniles or even 100 days as adult trees (Siebel et al., 1998; Kreuzwieser et al., 2004; Glenz et al., 2006). In years with spring flooding events, these trees may alter their wood anatomy and frequently form tree rings with anomalously narrow earlywood vessels – such rings are known as ‘flood rings’ (Astrade and Bégin, 1997; St. George et al., 2002; Tardif et al., 2010; Ballesteros-Cánovas et al., 2015; Therrell and Bialecki, 2015; Bräuning et al., 2016; Kames et al., 2016). These earlywood vessels may sometimes be accompanied by sickle-shaped, collapsed earlywood vessels (Land, 2014). When flooding occurs during summer, exceptionally large latewood vessels may occur (Yanosky, 1983; Yanosky and Cleaveland, 1998; Land, 2014). As flood rings are not only found in living trees but are also preserved in old timber and in subfossil trees, they can be used as a proxy to reconstruct flooding events with an annual or even intra-annual accuracy over potentially millennia and may shed light on the forcing factors between climate, human impact, and flooding events (Yanosky, 1983; Wertz et al., 2013; Land, 2014; Ballesteros-Cánovas et al., 2015; Kames et al., 2016). However, the application of flood rings as proxy for flooding events is hampered by our limited understanding of their formation, in the absence of experimental evidence (St. George, 2010).

The formation of flood rings is, *inter alia*, depending on the time window during which developing xylem cells are able to register the flooding signal (Fonti et al., 2010; Sass-Klaassen et al., 2011). Flooding events during winter dormancy are most likely not recorded whereas during the period of radial growth the flooding signal can be directly recorded in the earlywood (St. George and Nielsen, 2002; Wertz et al., 2013) or latewood (Yanosky, 1983; Land, 2014). In ring-porous species radial growth may either start during late bud dormancy or after budswell, while earlywood formation normally ends after the leaves are fully expanded (Zasada and Zahner, 1969; Bréda and Granier, 1996; Sass-Klaassen et al., 2011; Takahashi et al., 2013) and fine roots have developed (Ponti et al., 2004). Radial growth cessation is highly variable among trees and from year-to-year, and may end before leaf abscission (Michelot et al., 2012) or immediately after earlywood formation in spring (Land, 2014). Besides timing, the duration of a flooding event is also of importance (Astrade and Bégin, 1997; St. George and Nielsen, 2002; St. George, 2010). So far, it is known from flooding experiments that 6 weeks of flooding during leaf development can induce the formation of a flood ring in adult pedunculate oak (*Quercus robur* L.; Stuijfzand et al., 2008). Field studies also showed that flooding events of more than 10 days may induce flood rings in *Q. lyrata* and *Q. macrocarpa* (Therrell and Bialecki, 2015). Flooding height is less important, as 20 cm of flooding already induced flood rings in the submerged stem parts of pedunculate oak (Stuijfzand et al., 2008).

The physiology of flood-ring formation is poorly understood. During flooding, hypoxic conditions occur as gas diffusion rates are reduced by ∼10^-4^ in water compared to air (Cannon, 1925; Kozlowski, 1984). During the growing season, this may inhibit root growth and cause decay and dieback of roots, especially in non-woody fine roots (Coutts, 1982; Yamamoto and Kozlowski, 1987). The reduction of root biomass negatively influences root/leaf ratio and might be the key factor to explain reduced growth of flooded trees (Schmull and Thomas, 2000). Furthermore, reduced growth in flooded trees might occur as trees shift from aerobic respiration to anaerobic respiration which is much less efficient (Hook, 1984). Increased levels of hormones like ethylene and auxin in flooded stem parts have been related to morphological adjustments to cope with the effects of flooding, i.e., the enlargement of lenticels, formation of aerenchyma tissue and adventitious roots which deal with gas exchange and water uptake (Gomes and Kozlowski, 1980; Yamamoto et al., 1995; Parelle et al., 2006). These hormones could also be related to the formation of flood rings, as increased concentrations are associated with decreases in cross-sectional vessel areas and increases in vessel densities (Tuominen et al., 1995; Junghans et al., 2004; Aloni, 2013).

In this study, we experimentally investigated the formation of flood rings in pedunculate oak in relation to spring leaf-phenology and flooding durations lasting for 2, 4, and 6 weeks. We hypothesized that (i) ring-porous species that need their current year’s earlywood vessels for axial water transport (Cochard and Tyree, 1990; Tyree and Zimmermann, 2002; Copini et al., 2015), start earlywood-vessel development both in flooded and non-flooded stem parts; (ii) spring flooding leads to anomalously narrow earlywood vessels in flooded stem parts when the timing of flooding coincides with earlywood-vessel development within 6 weeks of flooding; (iii) based on the study of Land (2014), developing earlywood vessels collapse in response to flooding; and (iv) flooding leads to reduced radial growth and root dieback when flooding occurs during the growing season.

## Materials and Methods

### Plant Material

We used 200 four-year-old potted pedunculate oak trees (*Quercus robur* L.) with a stem height of approximately 180 cm, that were randomly selected out of 600 available trees. All 600 trees were obtained from a tree nursery in March 2009, 1 year before the experiment was conducted, and immediately potted in 17 l containers (diameter 30 cm, height 24 cm) in a sand-loam mixture. The trees were then placed in a 1 m × 1 m grid in an experimental garden in Wageningen, the Netherlands (51.9884°N, 5.6644°E). The trees were frequently watered using a semi-automatic fertigation system.

### Experimental Set-up

**FIGURE 1:**
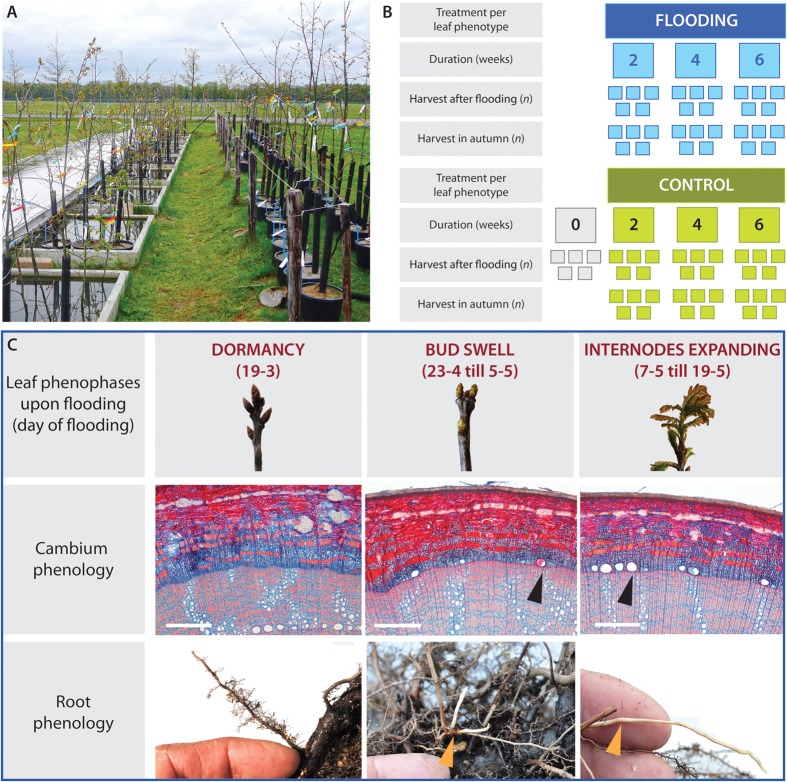
**Experimental set-up of the flooding experiment.** **(A)** Experimental set-up showing the concrete basins with experimentally flooded trees on the left and corresponding control trees on the right. **(B)** At three leaf phenophases, trees were flooded to a stem height of 50 cm for 2, 4, or 6 weeks (*n* = 10); control trees were placed next to treated trees. Upon budswell, an additional group of trees had only their roots flooded during 2, 4, or 6 weeks (*n* = 10). After the flooding period in spring, five trees per treatment were harvested immediately while the remaining trees were returned to the experimental garden to be harvested after the end of the growing season, in autumn. **(C)** Cambium and root phenophases in relation to the three leaf phenophases upon the onset of flooding. When the buds were dormant on March 19th, 2010, the cambium and roots were dormant as well with no earlywood vessels or white roots present. Upon budswell, between April 23rd and May 5th, earlywood-vessel development had started irregularly around the circumference. Normally, earlywood vessels were unlignified while in some cases lignification had started (arrow). In all trees, newly formed elongating white roots were present (arrow). When the internodes started expanding 14 days after budswell, between May 7th and May 19th, earlywood-vessel development had started in all trees and both lignified (arrow) and unlignified vessels were present and many new elongating white roots had formed.

The flooding experiment was conducted at the Sinderhoeve Research Station, Wageningen University, The Netherlands (51.9983°N, 5.7523°E) between March and July 2010. To simulate flooding, we used 1.4 m × 1.2 m × 0.7 m basins (length, width, depth) containing pumps for water re-circulation and maintaining a water level to flood trees to a stem height of 50 cm (**Figure 1**). The water came from a rain fed basin. We installed six two-channel HOBO Pro temperature data loggers (Onset Corporation, Bourne, MA, USA) to record water temperature 25 cm below the water surface (i.e., at 25-cm stem height) of every basin and air temperature 25 cm above the water surface, corresponding to 75-cm stem height of the flooded trees. During the experimental period, the mean water temperature of 15.2 ± 4.6°C (mean ± SD) was generally higher than the mean air temperature (13.1 ± 6.6°C) while the daily temperature amplitude (maximum temperature – minimum temperature) in water was much lower compared to the air temperature (**Supplementary Figure S1**). Below water, dissolved oxygen concentrations (mg/L) were recorded once a week using a portable WTW Oxi 330 meter, equipped with a CellOx 325 electrode. Oxygen concentrations were on average 10.6 ± 3.9 mg/l (mean ± SD). Differences in oxygen concentrations occurred both in time and between different basins (**Supplementary Figure S1**).

Flooding treatments started at three successive leaf phenophases (**Figure 1**) taking into account the leaf status of individual trees: late dormant trees were flooded on March 19th, trees with swelling buds were flooded between April 23rd and May 5th, and trees with expanding internodes were flooded between May 7th and May 19th, 2010, approximately 2 weeks after budswell (**Figure 1**). At each phenophase, 10 trees were flooded for either 2, 4, or 6 weeks by randomly placing them in one of the 10 basins (90 stem-flooded trees). For the budswell group, an additional treatment was added in which the roots of 10 trees were flooded for either 2, 4, or 6 weeks by leveling the water to the soil surface (30 root flooded trees; **Figure 1**). Control trees, corresponding to all flooding durations were placed next to the basins (**Figure 1**).

To assess the status of earlywood-vessel and root formation of the trees entering the experiment, five trees per phenophase were harvested (**Figure 1**). All control trees were watered twice a week and remained – like all flooded trees – exposed to ambient weather conditions. To study the dynamics of flood-ring development, we harvested half of the trees immediately after the flooding treatment (five treated trees, five control trees); the remainder was returned to the experimental garden and was harvested after the end of the growing season in November 2010. Stem sections were sampled at 25 cm (i.e., 25 cm below water level in the flooded trees) and 75-cm (i.e., 25 cm above water level) stem height. Samples were stored in 50% ethanol solution at room temperature prior to further processing.

### Wood Sample Preparation and Measurements

For both stem samples collected at flooding cessation and at the end of the growing season, transverse wood sections were cut with a thickness of approximately 20 μm using a G.S.L.-1 sliding microtome (Gärtner et al., 2014) and stained with a safranin/astra blue solution for 5 min. Following dehydration in graded series of ethanol (50–95–100%), samples were rinsed with xylol, mounted in Canada balsam and dried at 60°C for 15 h. Pictures were made with a digital camera (DFC 320, Leica, Cambridge, UK) mounted on a microscope (DM 2500, Leica, Cambridge, UK) using Leica imaging software (version 3.6.0).

For all trees harvested, we measured earlywood-vessel size (in μm^2^) over a tangential width of approximately 1 cm – which equals approximately 20% of the circumference of the 2010 tree ring – using ImageJ software (ver. 1.44^1^; developed by W. Rasband, National Institutes of Health, Bethesda, MD, USA). In order to get an estimate of vessel development in spring, we distinguished unlignified vessels (completely blue) from lignified vessels (partly or completely red). We then determined mean earlywood-vessel area, and calculated the maximum earlywood-vessel size as the mean of the 20 largest earlywood vessels. In addition, earlywood-vessel density (vessels/mm) was calculated by dividing the number of first-row earlywood vessels formed in 2010 over the tangential width (vessels/mm). The 2010 ring width was measured at two radii at both stem heights in the trees that were harvested in November. Collapsed vessels were visually detected per stem height and rings containing three or more collapsed vessels per stem height were recorded as tree rings containing collapsed vessels.

### Root Development

We recorded root phenophases of all trees that were harvested immediately after the flooding experiments. To do so, we removed the basal stem part with the roots attached from the container and rinsed it with water to remove the soil. Based on studies by Hendrick and Pregitzer (1992) and Ponti et al. (2004), we defined three root phenophases: (i) dormant roots (ii) white roots elongating, and (iii) white roots maturing, forming many small lateral roots. We recorded whether root formation was affected by root dieback as visible by black discolorations or further decay. In addition, the presence of lenticels and adventitious roots was noted.

### Statistical Analysis

All statistical analyses were performed in the statistical software package SPSS, version 19 (SPSS Inc., Chicago, IL, USA) applying a significance level of 0.05.

#### Earlywood-Vessel Development

We used the trees that were harvested in spring upon the termination of the flooding treatments, to test for differences between earlywood-vessel development below and above water level as compared to control trees. First, we tested for a possible difference in vessel density at 25-cm and 75-cm stem height between all flooded and control (grouping trees with different flooding durations) using a Mann–Whitney *U* test (*n* = 15). Subsequently, we performed three Mann–Whitney *U* tests, to assess whether significant differences occurred after 2, 4, or 6 weeks of flooding, compared to control trees (*n* = 5).

#### Earlywood-Vessel Area

The trees that were harvested at the end of the growing season were used to test whether spring flooding leads to anomalously narrow earlywood vessels when flooding coincides with earlywood vessel development, i.e., during budswell and to a lesser degree when the internodes start growing within 6 weeks of flooding. First, we used mixed factorial analyses of variance (ANOVA) to test whether mean and maximum earlywood-vessel area and vessel density within each treatment (control, flooded during late bud dormancy, budswell and upon internode expansion) was significantly different in relation to stem height (within subject factor) and flooding duration (between subject factor; *n* = 5). Second, to test for differences between flooding treatments and their control trees, we used mixed factorial ANOVAs with mean and maximum vessel size and vessel density at 25-cm (below water) and at 75-cm stem height (above water) as within-subject factors and flooding durations and treatment as between-subject factors (*n* = 5).

#### Vessel Collapse

We tested whether the occurrence of collapsed earlywood vessels was significantly different at 25-cm stem height between flooded and control trees by using the trees that were harvested after flooding and after the end of the growing season. First we used Fisher’s Exact tests to evaluate whether there was an significant effect between all flooded and control trees per phenophase (*n* = 15). Subsequently, we performed three separate Fisher’s Exact tests per phenophase to determine whether significant differences between flooded and control trees occurred after 2, 4, or 6 weeks (*n* = 5).

#### Radial Growth

To test whether flooded trees have significantly smaller ring widths in flooded stem parts, or along the whole stem because of the flooding treatments, we used ring widths of the trees that were harvested at the end of the growing season and used mixed factorial ANOVAs to test whether the ring widths at both 25 and 75 cm (within subject factor) was affected by the flooding treatment and flooding durations (between subject factors). *Post hoc* tests with Bonferroni corrections were conducted to assess the impact of flooding durations (2, 4, or 6 weeks) on radial growth.

#### Root Dieback

For roots, it was tested whether dieback occurred in trees that were flooded (stem or root flooded) using the trees that were harvested immediately after the end of the flooding treatments. First we used Fisher’s Exact tests to assess whether significant differences occurred between flooded and control trees per phenophase (*n* = 15). Subsequently, we performed three separate Fisher’s Exact tests per phenophase to determine whether significant differences between flooded and control trees occurred after 2, 4, or 6 weeks (*n* = 5).

## Results

### Earlywood-Vessel Development during Flooding

#### Late Bud Dormancy

At March 19th, when the first flooding treatment started, none of the trees had formed earlywood vessels (**Figure 1A**). Also, after 2 and 4 weeks of flooding none of the flooded and control trees had started earlywood vessel development (**Supplementary Figure S2**). The first earlywood vessels were present 6 weeks after the start of the flooding treatment in three flooded trees and in three control trees of which the buds were broken. In contrast to the control trees, that started earlywood-vessel development both at 25-cm and at 75-cm stem height, the flooded trees had started earlywood-vessel development only above the water level at 75-cm stem height (**Supplementary Figure S2**).

#### Budswell

**FIGURE 2:**
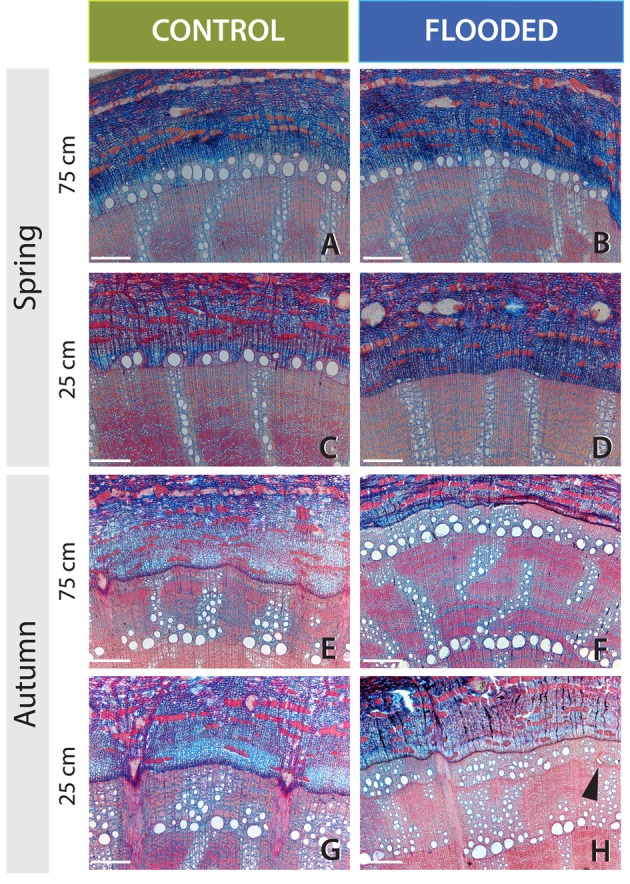
**Effects of 6 weeks of flooding after budswell on vessel development and radial growth in pedunculate oak.** The white scale bar represents 300 μm. The cambial zone of control (left) and flooded trees (right) at 25 cm (flooded) and 75 cm (non-flooded) immediately after the flooding treatment in Spring 2010 **(A–D)** or after the growing season in Autumn 2010 **(E–H)**. **(A,B)** The cambial zone at 75-cm stem height. In both trees many lignified earlywood vessels are present. **(C,D)** The cambial zone at 25-cm stem height of the same trees as in **(A,B)**. Whereas in the control tree vessel development started and many earlywood vessels are lignified in the flooded tree, 25 cm below water hardly any vessels have been formed and most are not yet lignified. **(E,F)** The cambial zone at 75-stem height (not submerged). In both the control and flooded tree the earlywood vessels are relatively large, while ring width in the flooded tree is strongly reduced. **(G,H)** The cambial zone at 25-cm stem height (submerged stem parts in flooding treatments). While the trees of the control group showed relatively large earlywood vessels and wide ring widths, the flooded trees formed on average 70% smaller vessel areas and 59% smaller tree rings.

Upon the second phenophase, between April 23rd and May 5th, 2010, earlywood-vessel development had started in all but one trees (**Figure 1C**). Earlywood vessels were initiated irregularly around the stem circumference, mostly near latewood vessels which were bordering the tree-ring boundary. In one tree, some earlywood vessels were lignified while in the others vessels were still unlignified. The flooding treatments induced significant differences in vessel densities between 25- and 75-cm stem height compared to the control trees (Mann–Whitney *U* test, *U* = 24, *P* < 0.001, *n* = 15; **Table 1**, **Figures 2A–D**). Two weeks of flooding did not lead to differences in vessel densities, but after 4 or 6 weeks significant differences occurred between flooded and control trees (Mann–Whitney *U* test, *U* = 0, *p* = 0.008, *n* = 5 and *U* = 2, *p* = 0.032, *n* = 5, respectively). After 6 weeks of flooding the second row of earlywood vessels was already being completed above the water level (**Figure 2B**, while below water vessel development was hampered (**Figure 2D**). In the trees of which the roots were flooded upon budswell, no significant effects of the treatment or of flooding duration were observed.

**Table 1:** Mean earlywood-vessel density and earlywood-vessel density differences and standard deviations (SD) between 75- and 25-cm stem height measured directly after the flooding treatments.

		Mean vessel density (#/mm, ±*SD*)			Treatment effect	
			
Phenophase	Treatment	25 cm stem height	75 cm stem height	Difference (75–25 cm)	*U*	*P*
Late bud Dormancy	Stem flooding	0 ± 0	0.25 ± 0.59	0.25 ± 0.59	NT	NT
	Control	0.19 ± 0.46	0.21 ± 0.62	0.01 ± 0.31		
Budswell	Stem flooding	1.97 ± 1.67	5.09 ± 1.32	3.12 ± 1.52	26	^∗∗∗^
	Control	3.48 ± 1.71	4.60 ± 1.84	1.13 ± 1.62		
Budswell	Root flooding	3.45 ± 1.78	5.07 ± 1.34	1.63 ± 1.39	102	–
	Control	3.48 ± 1.71	4.60 ± 184	1.13 ± 1.62		
Internode expansion	Stem flooding	3.51 ± 1.06	5.83 ± 1.31	2.32 ± 1.38	55	^∗^
	Control	4.85 ± 0.84	5.80 ± 1.17	0.95 ± 1.54		


*The trees that were flooded for 2, 4, and 6 weeks were pooled per phenophase (*n* = 15). The effect of flooding on the difference of earlywood-vessel density was tested using Mann–Whitney *U* tests. *U*-values and their significance are given as: ^∗^*P* < 0.05; ^∗∗^*P* < 0.01; ^∗∗∗^*P* < 0.001; NT, not tested.*

#### Internode Expansion

During the last phenophase, between May 7th and May 19th, earlywood-vessel development had started in all trees (**Figure 1C**). While some trees had just formed narrow unlignified earlywood vessels, others showed few (in one tree many) lignified vessels (**Figure 1C**). After this late flooding treatment significantly larger differences in vessel densities occurred between 25- and 75-cm stem height compared to the control trees (Mann–Whitney *U* test, *U* = 24, *P* < 0.05, *n* = 15) as in the flooded stem parts earlywood vessel development was slightly lower (**Table 1**). No significant differences were found in relation to flooding duration. At the end of the 6-week flooding treatment, i.e., 8 weeks after budswell, all earlywood vessels of the control trees were lignified both at 25- and 75-cm stem height. In contrast, in flooded trees only the earlywood vessel above the water level were lignified whereas below the water level many vessels remained unlignified.

### Earlywood Vessel Development during the Growing Season

#### Late Bud Dormancy

**FIGURE 3:**
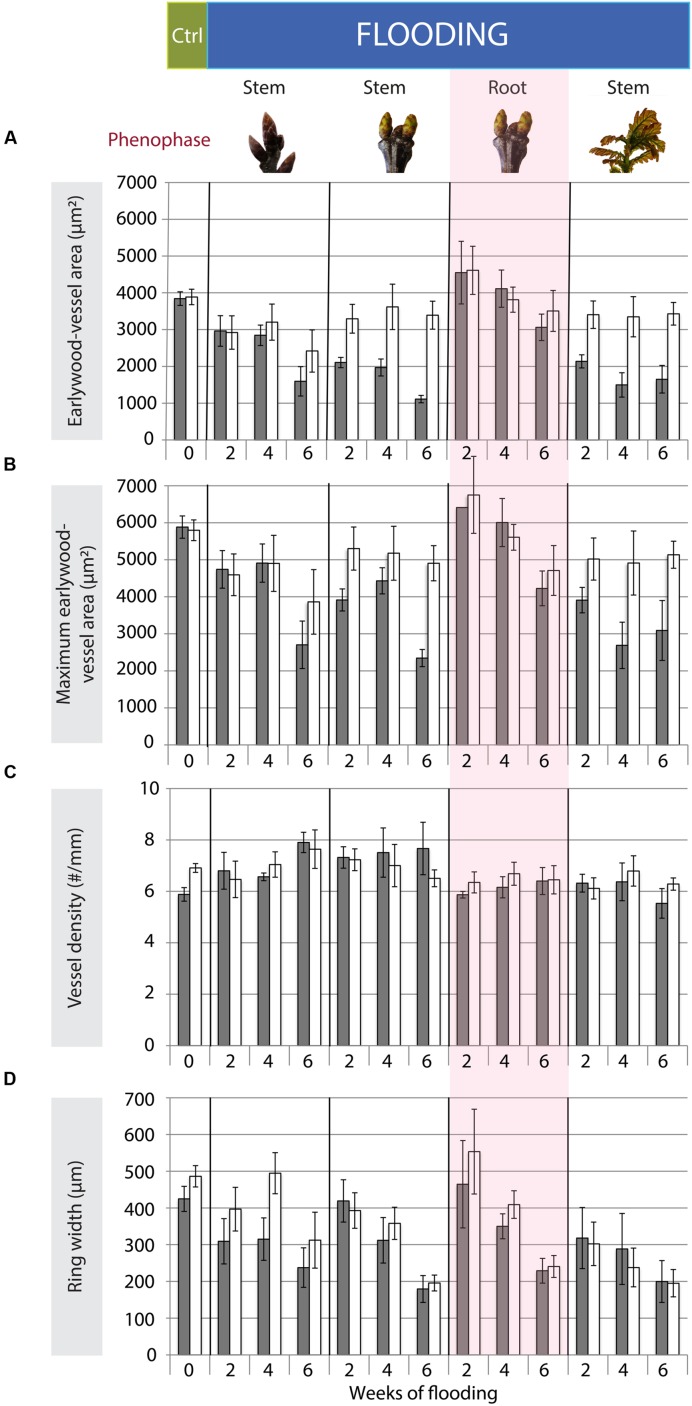
**Mean earlywood-vessel area (**A**: μm^2^), means of the 20 largest earlywood vessels (**B**: maximum earlywood-vessel area, μm^2^), vessel density (**C**: #/mm) and ring width (**D**: μm) after the end of the growing season following the flooding experiments for the different treatments (*n* = 5) and control trees (*n* = 35).** Gray bars show the mean values and standard errors of stem sections taken at 25-cm stem height; whereas white bars represent the mean and standard errors of the stem section at 75-cm stem height. The control trees were pooled as no significant differences occurred among them.

Trees that were flooded upon late bud dormancy had formed slightly narrower mean earlywood vessels at 25-cm compared to 75-cm stem height by the end of the growing season (**Table 2**, **Figure 3A**). This difference was mainly caused by the trees of the 6-week flooding treatment of which two had started with leaf formation after 4 weeks of flooding – within the 6-week flooding treatment – and showed a 38 and 66% reduction in earlywood-vessel size in flooded stem parts at the end of the growing season. Maximum earlywood-vessel size and vessel density were comparable at both heights (**Table 2**, **Figures 3B,C**, **Supplementary Figure S3**). Compared to control trees, mean earlywood-vessel area and maximum earlywood-vessel area were significantly reduced at both heights and vessel density was slightly higher, especially in the flooded stem part (**Table 3**, **Figure 3**). The significant interaction between duration and treatment was caused by the trees of which the buds started to develop during the flooding experiment. When the 6-week treatment – in which earlywood-vessel formation may have started – was omitted, there was no effect of the flooding treatments upon earlywood-vessel size or vessel density.

**Table 2:** Effects of flooding per phenophase on mean earlywood-vessel area (Mean vessel area μm^2^), mean of the 20 largest earlywood vessels (Maximum vessel area, μm^2^), vessel density (#/mm), and ring width (μm), measured after the end of the growing season following the flooding experiments.

	Stem height		Height		Duration	
			
Variables	25 cm	75 cm	*F*	*P*	*F*	*P*
**Control non-flooded (*n* = 35)**						
Mean vessel area	3842 ± 823	3887 ± 936	0.064	–	2.365	–
Maximum vessel area	5883 ± 1346	5798 ± 1247	0.073	–	1.744	–
Vessel density	5.89 ± 1.17	6.91 ± 0.77	13.732	^∗∗^	2.390	–
Ring width	425 ± 153	486 ± 130	9.551	^∗∗^	1.642	–
**Late bud dormancy stem flooding (*n* = 15)**						
Mean vessel area	2467 ± 997	2847 ± 1105	4.727	^∗^	1.832	–
Maximum vessel area	4118 ± 1554	4452 ± 1601	1.894	–	1.987	–
Vessel density	7.49 ± 1.66	6.91 ± 1.08	0.056	–	1.494	–
Ring width	287 ± 125	401 ± 154	11.448	^∗∗^	1.475	–
**Budswell stem flooding (*n* = 14)**						
Mean Vessel area	1797 ± 617	3422 ± 910	46.454	^∗∗∗^	1.653	–
Maximum Vessel area	3500 ± 1086	5124 ± 1180	23.886	^∗∗∗^	2.916	–
Vessel density	7.5 ± 0.17	6.91 ± 0.37	0.063	–	0.462	–
Ring width	303 ± 148	313 ± 120	0.170	–	8.904	^∗∗^
**Budswell root flooding (*n* = 14)**						
Mean vessel area	3862 ± 1295	3932 ± 1126	0.123	–	1.529	–
Maximum vessel area	5486 ± 1724	5614 ± 1610	0.365	–	2.485	–
Vessel density	6.16 ± 0.87	6.50 ± 0.98	0.753	–	0.242	–
Ring width	334 ± 162	390 ± 181	0.170	–	5.765	^∗^
**Internode expansion stem flooding (*n* = 15)**						
Mean vessel area	1761 ± 695	3394 ± 874	52.960	^∗∗∗^	0.319	–
Maximum vessel area	3230 ± 1393	5023 ± 1316	36.223	^∗∗∗^	0.336	–
Vessel density	6.07 ± 1.2	6.40 ± 0.39	0.753	–	0.384	–
Ring width	269 ± 175	245 ± 114	0.581	–	0.841	–


*The mean and SD and the difference as a percentage are provided for each variable at 25- and 75-stem height. In addition, the table shows the results of mixed factorial ANOVAs with height as a within subject measure and flooding duration as a between-subject factor. *F-*values and their significance are provided as: ^∗^*P* < 0.05; ^∗∗^*P* < 0.01; ^∗∗∗^*P* < 0.001. The interactions of height and flooding duration were always insignificant (not shown).*

**Table 3:** Effects of flooding measured after the end of the growing season following the flooding experiments, on mean earlywood-vessel area (mean vessel area, μm^2^), mean of the 20 largest earlywood vessels (maximum vessel area, μm^2^), vessel density (#/mm) and ring width (μm) compared to control trees.

	Treatment		Duration		Duration ^∗^ treatment		Height		Height ^∗^ duration		Height ^∗^ treatment	
						
Variables	*F*	*P*	*F*	*P*	*F*	*P*	*F*	*P*	*F*	*P*	*F*	*P*
**Late bud dormancy stem flooding versus control^∗^**								
Mean vessel area	2.771	–	1.155	–	1.704	–	0.002	–	0.157	–	0.681	–
Maximum vessel area^∗^	0.695	–	0.606		1.576	–	0.695	–	0.173	–	0.312	–
Vessel density^∗^	0.347	–	1.260	–	0.617	–	1.699	–	0.164	–	1.238	–
Ring width^∗^	3.778	–	0.917	–	0.659	–	17.155	^∗∗∗^	2.638	–	0.793	–
**Budswell stem flooding versus control**								
Mean vessel area	25.684	^∗∗∗^	0.587	–	5.016	^∗^	27.123	^∗∗∗^	2.995	–	22.639	^∗∗∗^
Maximum vessel area	20.462	^∗∗∗^	0.612	–	5.673	^∗^	8.603	^∗∗^	2.605	–	10.913	^∗∗^
Vessel density	7.029	^∗^	0.667	–	1.030	–	1.740	–	1.004	–	3.153	–
Ring width	15.853	^∗∗^	8.755	^∗∗^	1.282	–	6.165	^∗^	1.478	–	3.287	–
**Budswell roots flooding versus control**								
Mean vessel area	0.145	–	1.618	–	2.917	–	0.227	–	2.101	–	0.001	–
Maximum vessel area	0.096	–	1.697	–	4.099	^∗^	0.012	–	1.183	–	0.265	–
Vessel density	0.172	–	1.591	–	1.056	–	7.658	^∗^	0.131	–	2.385	–
Ring width	3.703	–	8.061	^∗∗^	1.081	–	9.945	^∗∗^	0.652	–	0.348	–
**Internode expansion stem flooding versus control**								
Mean vessel area	23.217	^∗∗∗^	0.866	–	2.957	–	36.450	^∗∗∗^	1.685	–	29.277	^∗∗∗^
Maximum vessel area	14.826	^∗∗∗^	0.352	–	2.026	–	22.345	^∗∗∗^	1.856	–	18.937	^∗∗∗^
Vessel density	0.637	–	0.453	–	2.083	–	9.619	^∗∗^	1.258	–	2.208	–
Ring width	11.366	^∗∗^	0.195	–	0.866	–	1.389	–	0.864	–	6.118	^∗^


*The table shows the results of mixed factorial ANOVAs in which height was the within-subject measure and flooding treatment (treatment vs. control) and flooding duration were between subject factors. *F-*values and the *P*-values are given as: ^∗^*P* < 0.05; ^∗∗^*P* < 0.01; ^∗∗∗^*P* < 0.001. ^∗^Note that the 6-week group (late bud dormancy) was not analyzed as these trees started with budswell and leaf formation.*

#### Budswell

One tree that was flooded upon budswell died after the 4-week flooding treatment and was excluded from analyses. The remaining trees contained anomalously narrow earlywood vessels in the flooded stem parts at the end of the growing season; mean earlywood-vessel area and maximum earlywood-vessel area were significantly reduced on average by 47 and 32%, respectively, compared to 75-cm stem height, independent of flooding duration (**Table 2**, **Figures 2E–H** and **3A,B**, **Supplementary Figure S3**). Earlywood-vessel density was comparable between 25- and 75-cm stem height (**Table 2**, **Figure 3C**). Compared to the control trees, the flooded trees contained significantly lower mean or maximum earlywood-vessel areas, and higher vessel densities (**Table 3**, **Figure 3C**). In addition, the highly significant interactions for mean and maximum earlywood-vessel size between height and treatment, show that earlywood-vessel size was significantly reduced in the flooded stem parts (**Table 3**, **Figures 3A,B**).

One tree of the root flooding treatment (2 weeks) died and was excluded from the analyses. The remaining trees did not show any effect of root flooding on mean or maximum earlywood-vessel size, earlywood-vessel density in relation to stem height or flooding duration (**Table 2**, **Figure 3**, **Supplementary Figure S3**). Compared to the control trees, we found no effect of the root flooding treatment or flooding duration on mean or maximum earlywood-vessel area or vessel density (**Table 3**). Only a slightly significant interaction was observed in maximum vessel area between duration and treatment.

#### Internode Expansion

At the end of the growing season all trees that were flooded upon internode expansion, contained significantly narrower earlywood vessels (mean and maximum earlywood-vessel area) at 25-cm stem height compared to 75-cm stem height (**Table 2**, **Figures 3A,B**, **Supplementary Figure S3**) independent of flooding duration. On average, earlywood vessels areas were 50% smaller in the flooded stem parts compared to 75-cm stem height (**Table 2**). Vessel density was comparable at both heights (**Table 2**, **Figure 3C**). Compared to the control trees, the flooded trees contained significantly lower mean and maximum earlywood-vessel areas (**Table 3**, **Figures 3A,B**). In addition, we found highly significant interactions for earlywood-vessel size between height and treatment, indicating that earlywood-vessel size was significantly reduced in the flooded stem parts (**Table 3**, **Figures 3A,B**).

### Collapsed Vessels

**FIGURE 4:**
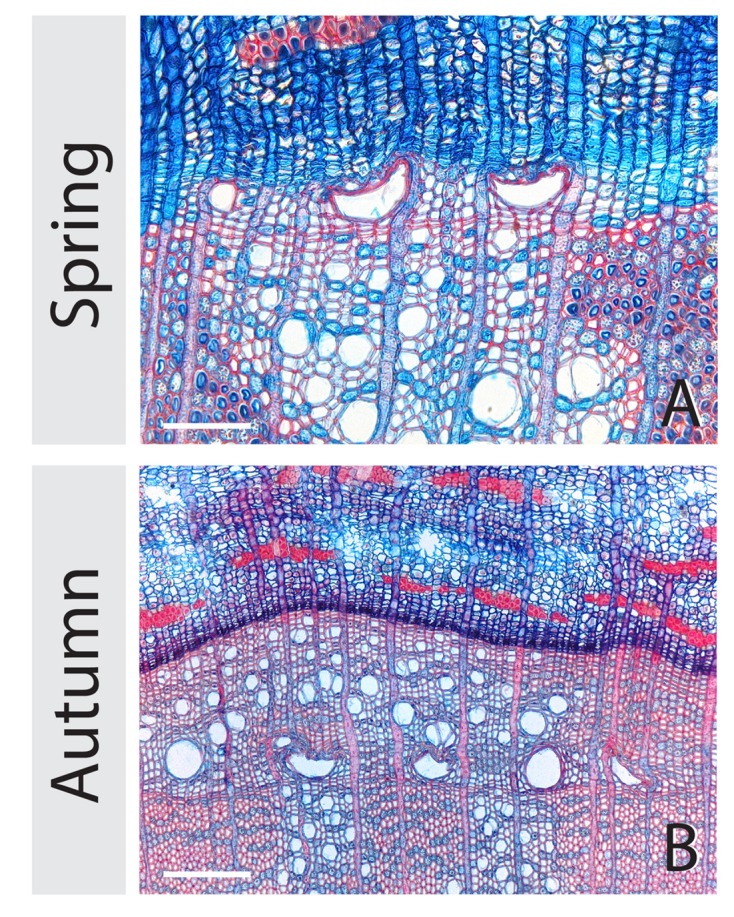
**Collapsed sickle shaped earlywood vessels in flooded stem part at 25-cm stem height.** Below the water level many irregularly shaped vessels are present both immediately after the flooding event **(A)** and after the end of the growing season **(B)**. The white scale bars represents 75 and 150 μm for **(A,B)**, respectively.

We found collapsed, sickle shaped earlywood vessels (**Figure 4**) in flooded stem parts (25-cm stem height) that were flooded upon the phenophases budswell and internode expansion. The number of trees containing collapsed earlywood vessels significantly differed between control and flooded trees after budswell, both immediately after flooding (Fisher’s Exact test, *p* = 0.006, *n* = 15) as well as after the growing season (Fisher’s Exact test, *p* = 0.017, 15 control trees, 14 flooded trees), Immediately after the flooding experiments, six out of 15 flooded trees contained collapsed vessels, whereas after the end of the growing season seven out of 15 trees were affected by vessel collapse. In the trees that were flooded after internode expansion, vessel collapse occurred in 11 (out of 15) trees immediately after the flooding experiments and was absent in control trees (Fisher’s Exact test, *p* < 0.001, *n* = 15) and in 14 (out of 15) after the end of the growing season and did not occur in control trees (Fisher’s Exact test, *p* < 0.001 *n* = 15). Flooding duration did not affect vessel collapse as highly significant differences (Fisher’s Exact test, *p* < 0.01 *n* = 5) were always observed after 2, 4, and 6 weeks in trees that were flooded upon bud swell or internode expansion; both immediate after the flooding treatments as at the end of the growing season.

### Root Development

#### Late Bud Dormancy

When the flooding treatment on dormant trees started, root formation had not yet started. The first white, elongating roots were present in four control trees belonging to the 6-week treatment, whereas in the flooded trees new root formation was absent (**Figure 1C**). One tree formed hypertrophied lenticels just below the water level during late bud dormancy.

#### Budswell

**FIGURE 5:**
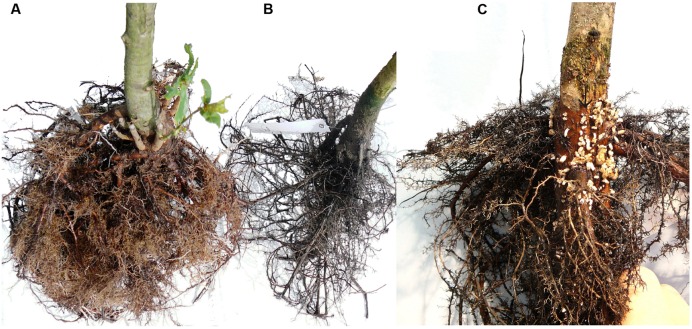
**The effect of the 6-week flooding treatment on root development of pedunculate oak that started at the phenophase budswell.** **(A)** A control tree with many maturing roots. **(B)** The root system of a tree that was flooded to a stem height of 50 cm; elongating or maturing roots are absent. **(C)** The root system of a tree of which only the roots were flooded; elongating or maturing white roots are absent but many hypertrophied lenticels have developed just below the water surface.

At budswell the formation of white roots in the elongation phase had started in all sampled trees (**Figure 1C**). We found significant differences in root dieback between all control and flooded trees (Fisher’s Exact test, *p* < 0.001). During the course of the experiment, root formation of control trees progressed from root elongation (after 2 and 4 weeks), to roots maturing after 6 weeks (**Figure 5A**) and no root dieback occurred. In contrast, in the flooded trees white roots in the elongation phase were unaffected by dieback after 2 weeks of flooding. However, after 4 weeks all white roots in the elongation phase were dying back (Fisher’s Exact test, *p* = 0.011) and after 6 weeks under water, roots were mostly decayed and easily detached from the main root system (Fisher’s Exact test, *p* = 0.008; **Figure 5B**).

Trees of which the roots were flooded, showed a similar pattern with significant root dieback occurring only in flooded trees (Fisher’s Exact test, *p* < 0.002). Significant differences between flooded trees and control trees occurred after 4 weeks (Fisher’s Exact test, *p* = 0.008) and 6 weeks (Fisher’s Exact test, *p* = 0.008). After 6 weeks most roots of flooded tress were decayed (**Figure 5C**). In contrast to the trees of which the stem was flooded, nine (out of 15) of the root flooded trees formed hypertrophied lenticels (**Figure 5C**).

#### Internode Extension

Trees that were flooded upon the phenophase internode extension, showed a similar pattern in root dieback (Fisher’s Exact test, *p* < 0.001). The roots of control trees progressed from roots in the elongation phase, after 2 weeks, to roots maturing after 4 and 6 weeks whereas their flooded counterparts were all affected by root dieback after 4 (Fisher’s Exact test, *p* = 0.008) and 6 weeks (Fisher’s Exact test, *p* = 0.008).

### Radial Growth

The mean ring widths were always significantly reduced in flooded trees as compared to control trees, when flooding occurred upon budswell or internode expansion (**Table 3**, **Figure 3D**). The trees that were flooded upon budswell showed a significant reduction in ring width in relation to flooding duration between the 2- and 6-week treatment (Bonferroni *Post hoc* test, *p* = 0.005) corresponding to mean ring widths of 4.06 and 1.88 mm, respectively (**Figure 3D**). Root flooding showed a similar patterns with reduced growth with increasing flooding duration; mean ring width was significantly smaller after 6 weeks of flooding compared to the 2-week treatment (Bonferroni *Post hoc* test, *p* = 0.018; **Table 2**, **Figure 3D**). In control trees and trees that were flooded during late bud dormancy, ring width was normally larger at 75-cm compared to 25-cm stem height. In flooded trees upon budswell or internode extension the effect of height was insignificant (**Table 2**, **Figure 3D**).

## Discussion

### Earlywood-Vessel Development is Suppressed in Flooded Stem Parts

In 4-year-old pedunculate oak trees harvested immediately after the flooding treatments earlywood-vessel development was suppressed in submerged stem parts if flooding occurred at budswell or internode expansion. In the two trees that were flooded during leaf dormancy but had started leaf development while flooded, vessel development was totally absent in flooded stem parts. This local impediment of earlywood-vessel development in flooded stem parts has, to the best of our knowledge, never previously been reported and is most likely caused by the hypoxic conditions accompanied with flooding (Kozlowski, 1984). As narrow earlywood vessels were frequently found lignified directly after the end of the flooding treatments, it seems likely that lignification occurs under anoxic conditions during flooding. The fact that earlywood-vessel development virtually stops in flooded stem parts is remarkable as ring-porous species need to form new earlywood vessel to replace the dysfunctional earlywood in the previous tree ring before the leaves are fully expanded (Cochard and Tyree, 1990; Tyree and Zimmermann, 2002; Copini et al., 2015). In case when no new earlywood vessels are formed in flooded stem parts, water transport most likely occurs in previous-year latewood vessels that are also thought to be of importance during spring reactivation (e.g., Utsumi et al., 1999; Tyree and Zimmermann, 2002; Copini et al., 2015).

### Flooding Reduces Earlywood-Vessel Size

We found that stem flooding significantly reduces mean and maximum earlywood-vessel area – on average by 50% – in flooded stem parts, when flooding occurs after budswell or internode expansion. This is independent of flooding duration (2, 4, or 6 weeks). The finding that already a 2-week flooding period strongly reduces earlywood-vessel size is in line with Therrell and Bialecki (2015) who studied flood rings in *Q. lyrata* and *Q. macrocarpa* along the Lower Mississippi River and linked flood rings to streamflow data. They reported that flooding events of more than 10 days during spring most likely induce a flood ring. Kames et al. (2016) also reported on the formation of flood rings in 2-year-old *Fraxinus pennsylvanica* trees, when experimental flooding for 3 weeks occurred during the period of earlywood development. Our results are moreover in accordance with many studies that show that earlywood formation normally only lasts between fourth and eighth weeks during spring (Sass-Klaassen et al., 2011; Gonzalez-Gonzalez et al., 2013).

By comparing stem with root flooding, we showed that stems need to be actually flooded to induce changes in the anatomy of tree rings. This is in line with results gained from a flooding experiment in which mature pedunculate oak trees were flooded till a stem height of 20 cm with stagnant water, resulting in a flood ring in the flooded stem part (Stuijfzand et al., 2008). In addition it may explain why St. George et al. (2002) found that flood rings were present in bur oak (*Q. macrocarpa* Michx.) at 45 cm, but were absent sometimes above 1.1 m and in general above 3 m. Also Land (2014) found that flood rings in *Q. robur* were present in flooded basal stem parts but absent at 4-m stem height.

Based on differences found between earlywood-vessel density recorded directly after flooding and after the end of the growing season (**Tables 1** and **2**), our results indicated that a substantial number of earlywood vessels must have been formed after the actual flooding events. These earlywood vessels did not enlarge to normal size, as in the control trees, even though they were not directly affected by anoxic conditions. We can only speculate on the processes behind the reduction of earlywood-vessel sizes after flooding has ceased. Possibly, earlywood-vessel enlargement after flooding was affected by high concentrations of auxin and ethylene which are known to increase during flooding events (Gomes and Kozlowski, 1980; Tang and Kozlowski, 1984; Aloni, 2013).

We expected that juvenile trees flooded after their internodes were expanding, would already contain many enlarged and lignified earlywood vessels, and that consequently mean earlywood-vessel area would be less reduced compared to flooding after budswell. This was not the case in our experiment. A possible reason could be that temperatures during the first 19 days of May 2010 were far below average and among the coldest measured since weather records of the Royal Netherlands Meteorological Institute (KNMI) began. We suspect that these adverse temperatures during the experiment had strongly slowed down earlywood-vessel development, while having less effect on the ongoing leaf development.

### Collapsed Vessels May Pinpoint Flooding Events

We frequently observed vessel collapse in flooded stem sections of many trees flooded upon the phenophase budswell or internode expansion. Earlywood vessel collapse in response to flooding has been reported in pedunculate oak trees growing along the river Main in Germany as well as in a experimentally flooded juvenile trees (Land, 2014). Also freezing temperatures during the period of vessel formation may induce vessel collapse and consequent formation of ‘frost rings’ (Stahle, 1990; Leuschner and Schweingruber, 1996; Bräuning et al., 2016). However, in contrast to collapsed vessels in frost rings, vessels observed in this study were not surrounded by callus tissue. As collapsed vessels were absent in control trees, and no callus tissue occurred, we can exclude frost as a triggering factor. However, as most trees in the budswell or internode-expansion phase had already started vessel formation prior to the flooding treatments, we assume that collapsed vessels are the result of vessel-development interruption during the expansion phase (Zasada and Zahner, 1969; Leuschner and Schweingruber, 1996) when the flooding events occurred. As this phenomenon only occurred in submerged stem sections after budswell and internode expansion, the presence of collapsed vessels in a tree ring can be used as a characteristic feature to pinpoint flooding events to the restricted period in the season when earlywood vessels develop.

### Radial Growth and Root Dieback

We found that flooding up to 50-cm stem height as well as root flooding reduces mean ring width when flooding occurred after budswell or internode expansion. This is in line with many studies on juvenile trees that showed that radial growth can be seriously hampered by spring or summer flooding (Coutts, 1982; Yamamoto and Kozlowski, 1987). The presence of many hypertrophied lenticels upon root flooding, which are permeable to water (Groh et al., 2002) and might play an important role in water supply during flooding events (Parelle et al., 2006), did not affect the tendency of trees to grow less with increasing flooding duration.

In our experiment, root dieback occurred in all trees that were flooded (stem or roots) for 4 or 6 weeks after budswell or internode expansion. Reduced growth after flooding is most likely related to the inhibited root development. This is in line with the general view that low oxygen concentrations may inhibit root initiation and seriously affect root development so that new roots need to be developed after flooding events (e.g., Coutts, 1982; Tang and Kozlowski, 1984; Siebel et al., 1998). In addition, reduced radial growth in flooded trees could be an effect of flooding-induced local reductions in earlywood-vessel size in basal stem parts, which creates a hydraulic bottleneck. Other studies explain reduced radial growth by reduced leaf area, stomata closure and early-leaf senescence occurring during flooding (Kozlowski and Pallardy, 1984; Schmull and Thomas, 2000). Whereas, stomata closure may have influenced growth in our experiment, leaf senescence did not occur in our study. Mature trees can tolerate flooding events better than juvenile trees (Kozlowski, 1984), which may partly explain why tree-ring width in mature riparian trees is not reduced in years with flooding events (Astrade and Bégin, 1997; Stuijfzand et al., 2008; Tardif et al., 2010; Land, 2014).

### Implications for Flood Reconstructions

In this study, we synchronized the timing of the flooding for all trees by initiating the flooding treatment at a specific leaf-phenological stage. Under natural flooding conditions, trees in forests can be in many different stages of leaf and xylem development. Consequently, a flood might be recorded in a particular tree, while the flooding signal is absent in other trees. This is in line with observations by St. George and Nielsen (2000), who found flood rings in between 6 and 24% of the sampled *Q. macrocarpa* trees. However, it should be noted that *Q. macrocarpa* is not a true riparian species, it normally grows on the upper floodplain terraces where trees are less frequently flooded. In contrast, Kames et al. (2016) working with riparian black ash (*Fraxinus nigra* Marsh.) trees found flood rings to be a common feature among trees. The authors also observed that the reduction in the mean vessel areas in flood rings was positively associated with flood intensity making continuous vessels area chronologies a proxy for flood duration. In line with studies from St. George et al. (2002), Stuijfzand et al. (2008), and Land (2014), we showed that sampling trees for flood signals should include the stem base as flood rings were observed to occur only in flooded stem parts. Potentially this means that by sampling at different heights, an estimation of the flood level might be retrieved. Further studies on mature trees in riparian forests are necessary to support this statement.

In congruence with reports from previous studies (e.g., Therrell and Bialecki, 2015; Kames et al., 2016), we found that relatively short floods, lasting for 2 weeks, induced the formation of narrow earlywood vessels in flooded stem parts when flooding occurs during earlywood formation. Since the earlywood vessels of ring-porous species are formed within a short time window (e.g., Sass-Klaassen et al., 2011; Gonzalez-Gonzalez et al., 2013), flood rings encode for flooding events that occur during a narrow time window. While the start and end of a flooding event does not seem relevant, the event must take place, or start in, the narrow period of earlywood formation. Collapsed vessels can be used to pinpoint flooding events that had started at the moment of earlywood-vessel development. These results indicate that flood reconstructions using flood rings may only be valid to floods that occurred in spring, i.e., at the onset of vessel formation.

## Conclusion

We conclude that relatively short periods of flooding (2, 4, and 6 weeks) reduces earlywood-vessel size drastically, on average by 50%, in flooded stem parts of juvenile pedunculate oak trees. This flood marker occurs in the growing season when the flooding event takes place, but only if flooding occurs after budswell or internode expansion when earlywood vessels are developing. As during flooding, earlywood-vessel development is hampered, the narrow earlywood vessels in flood rings consist of cells that are mainly formed after the actual flooding events. By comparing stem and root flooding, we demonstrated that flood rings only occur in trees of which the stem is flooded. Our study indicates that root dieback, together with strongly reduced hydraulic conductivity due to extremely narrow earlywood vessels in flooded stem parts, most likely contributes to reduced radial growth along the whole stem after flooding of juvenile oak trees.

## Author Contributions

PC, JdO, WL, and US-K designed the study. PC, WL, and LG conducted the research. PC and WL carried out the anatomical and statistical analyses and wrote the first draft of the manuscript which was intensively edited by all authors.

## Conflict of Interest Statement

The authors declare that the research was conducted in the absence of any commercial or financial relationships that could be construed as a potential conflict of interest.

## Footnotes

1 http://rsb.info.nih.gov/ij
