# Supplementary material for: Flood-Ring Formation and Root Development in Response to Experimental Flooding of Young Quercus robur Trees
Source: Front Plant Sci. 2016 Jun 14;7:775. doi: 10.3389/fpls.2016.00775 (PMC4906004; doi:10.3389/fpls.2016.00775)
Supplement: Supplementary file 1 [file Image_1.PDF]

Supplementary Material

Flood-ring formation and root development in response to experimental flooding of young *Quercus robur* trees.

Paul Copini*, **Jan den Ouden, Elisabeth M. R. Robert, Jacques C. Tardif, Walter Loesberg, Leo Goudzwaard, Ute Sass-Klaassen**

*** Correspondence:** Paul Copini: Paul.copini@wur.nl

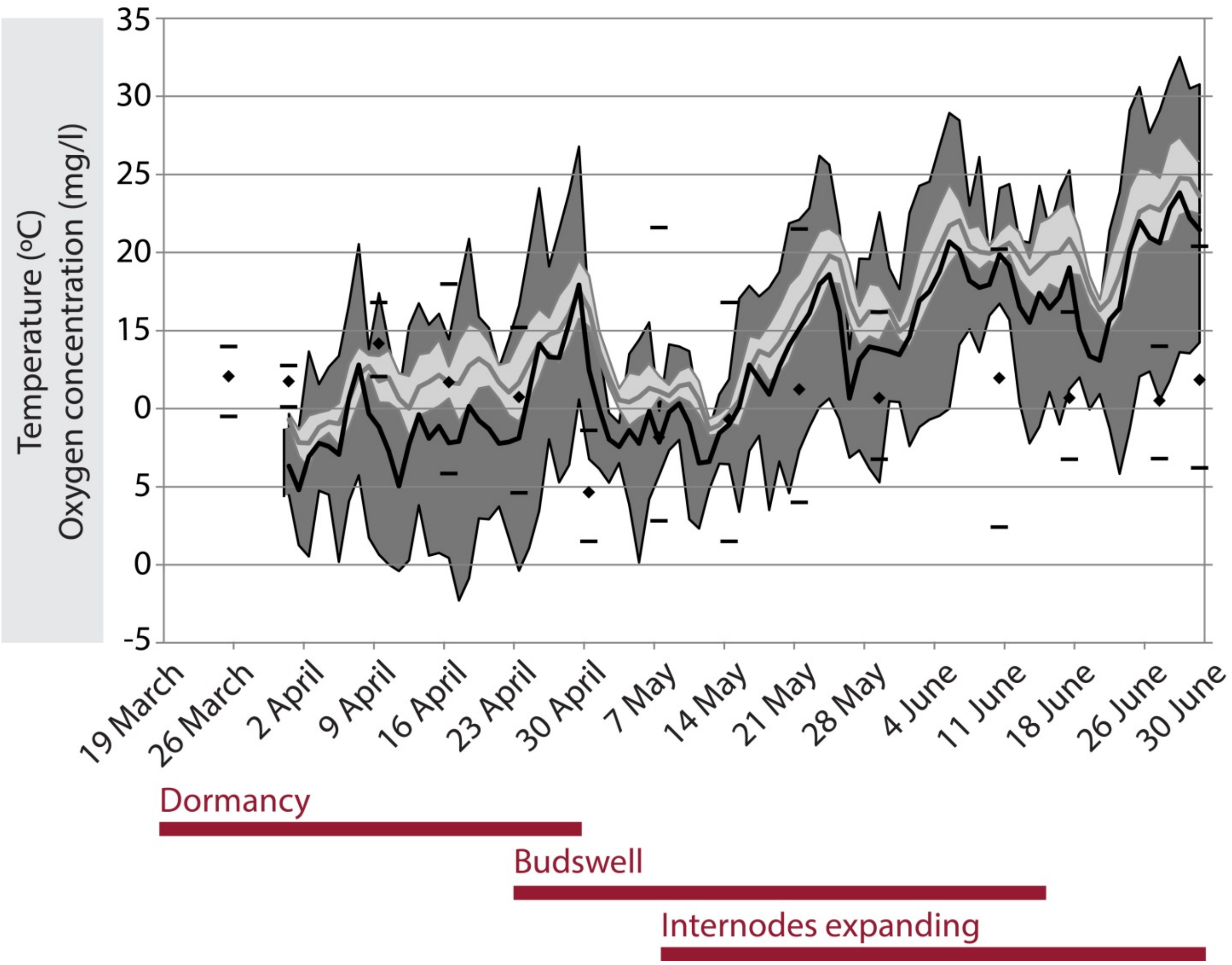

Supplementary Figure 1. Air and water temperature in combination with oxygen concentrations measured during the flooding experiments. Mean air temperature was measured 25 cm above the water level and is indicated by the black line, whereas the dark grey area represents the daily range. Mean water temperature was measured 25 cm below the water surface and is indicated by the grey line and the light grey area indicates the daily range in water temperatures. The mean oxygen levels (mg/l) are indicated with the diamond and the range is indicated with the horizontal lines. During the flooding treatments that started at the phenophase dormancy at March 19th and lasted till April 30th, the water and air temperature were 12.0 ± 2.6 °C and 9.6 ± 5.7 °C (mean ± SD), respectively. Note that we were unable to measure temperature during the first 11 days of the experiment. During the treatments that started upon budswell and were conducted between April 23rd and June 28th, the mean water and air temperature were 16.4 ± 4.2 °C and 14.5 ± 6.2 °C, respectively. In the last series of treatments that started on internode expansion, between May 7th and June 30th, the mean water and air temperature were 17.4 ± 4.4 °C and 15.5 ± 6.2°C respectively. Oxygen concentrations were highest in the period in which trees were flooded during late bud dormancy, with mean values of 12.4 ± 2.0 mg/l (mean ± SD). During the period when trees were flooded upon budswell or internode expansion oxygen concentrations were lower and the variation was higher with 9.8 ± 4.4 and 10.5 ± 4.1 mg/l respectively.

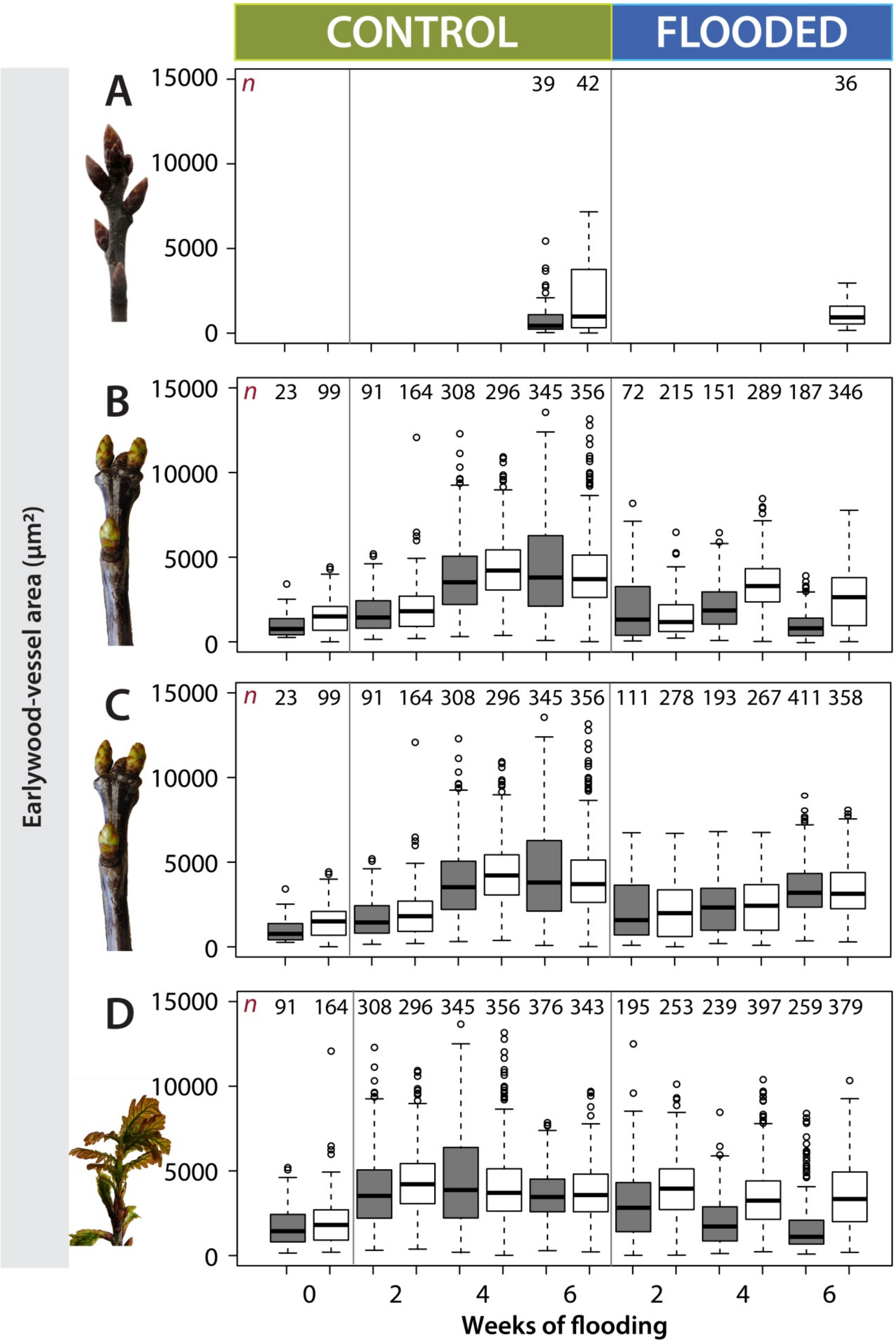

CONTROL
FLOODED
A
B
C
D
n
39 42 36
23 99 91 164 308 296 345 356 72 215 151 289 187 346
23 99 91 164 308 296 345 356 111 278 193 267 411 358
91 164 308 296 345 356 376 343 195 253 239 397 259 379
15000
10000
5000
0
Earlywood-vessel area (µm²)
0 2 4 6 2 4 6
Weeks of flooding

Supplementary Figure 2. Boxplots showing the vessel lumen areas (μm^2) of all measured earlywood vessels at 25-cm (gray boxes, flooded stem part) and at 75-cm (white boxes, non-flooded stem part) stem height. Above the boxplots the number of earlywood vessels is indicated. On the left, the control trees measured at time of treatment inititation (0 weeks) or immediately after the flooding treatment are shown and on the right all flooded trees are presented. Note that the same sets of control trees were used for different treatments. A: Stem flooding during late bud dormancy. After six weeks three trees had started earlywood vessel development of which one had started with leaf formation. Flooded trees only formed earlywood vessels above the water level, whereas the control trees formed earlywood vessels both at 25 and at 75cm. B: Stem flooding started upon budswell. Earlywood-vessel development occured above the water level at 75cm, while the vessels were narrow and less abundant in the submerged stem parts. C: Root flooding started on budswell. Earlywood-vessel development occured at both 25-cm and at 75-cm stem height. D: Stem flooding started upon internode expansion, approximately two weeks after budswell. Earlywood-vessel development above water was comparable to control trees. However below water, vessel development was hampered and vessels were more narrow.

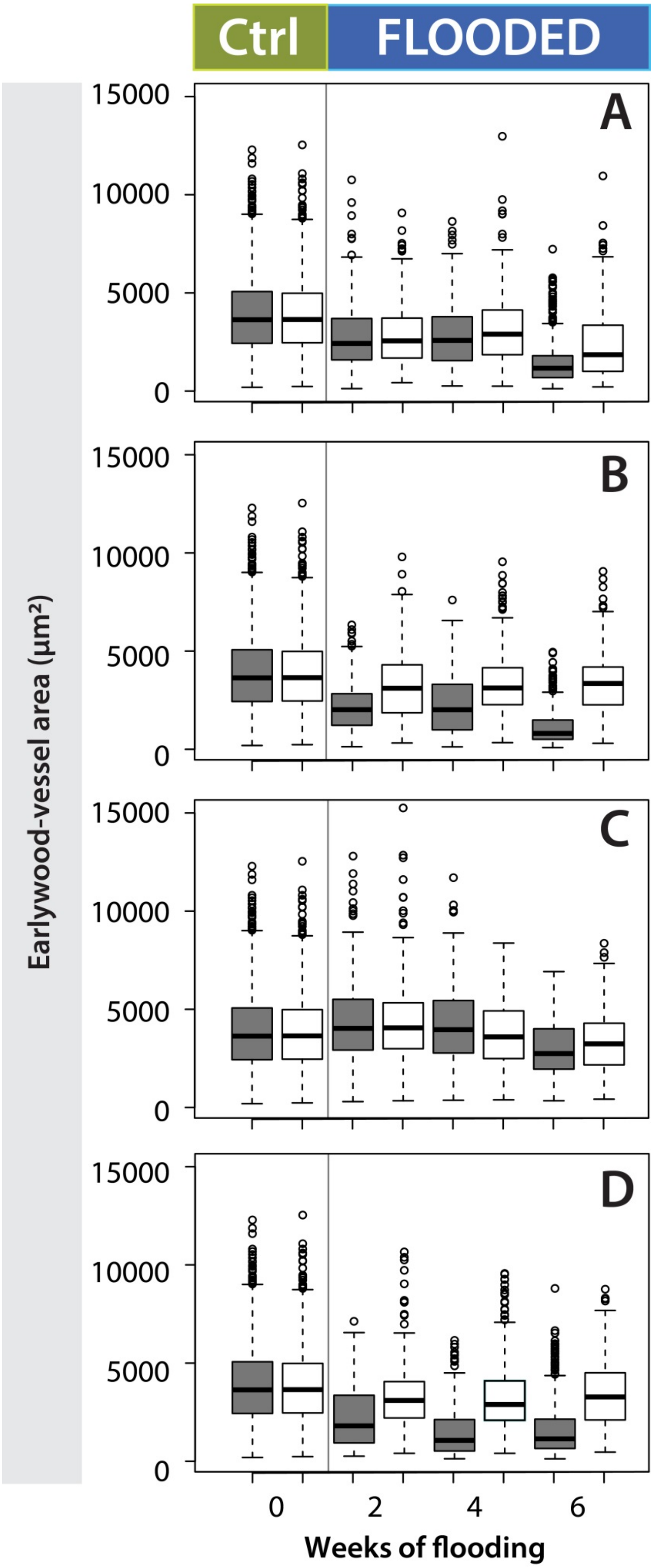
Ctrl
FLOODED
15000
10000
5000
0
A
B
C
D
Earlywood-vessel area (µm²)
0
2
4
6
Weeks of flooding

Supplementary Figure 3. Boxplot showing the vessel lumen areas (μm^2) of all measured earlywood vessels at 25-cm (gray boxes, flooded stem part) and at 75-cm (white boxes, non-flooded stem part) stem height that were formed during the growing season. Per tree on average 69 ± 18 vessels (mean ± SD) were measured. The control trees were pooled as no significant differences occured among them (n = 35). The other treatments represent five trees but note that after four weeks of stem flooding upon budswell one tree died just as after two weeks of root flooding. (A) Earlywood-vessel areas of trees that were flooded during late bud dormancy. On average the vessels were slightly narrower compared to the controls. After six weeks of flooding earlywood-vessel lumen area decreased below water level mainly because two trees had started leaf formation while flooded. (B) Flooding started on budswell. Earlywood-vessels area was strongly reduced in all flooding durations. (C) Root flooding started upon budswell. Earlywood-vessel lumen area was not significantly different compared to control trees. (D) Flooding started on internode expansion approximately two weeks after budswell. Earlywood-vessel were always narrower below water level.
